# Coronary Atherosclerosis: Causes, Consequences, and the Passage of Time∗

**DOI:** 10.1016/j.jacadv.2023.100756

**Published:** 2023-12-19

**Authors:** Peter P. Toth, Allan D. Sniderman

**Affiliations:** a Department of Preventive Cardiology, CGH Medical Center, Sterling, Illinois, USA; b Ciccarone Center for the Prevention of Cardiovascular Disease, Johns Hopkins University School of Medicine, Baltimore, Maryland, USA; c Department of Medicine, Mike and Valeria Rosenbloom Centre for Cardiovascular Prevention, McGill University Health Centre, Montreal, Quebec, Canada

**Keywords:** albuminuria, apolipoprotein B, atherosclerosis, cardiovascular risk, coronary artery calcification

**Figure:**
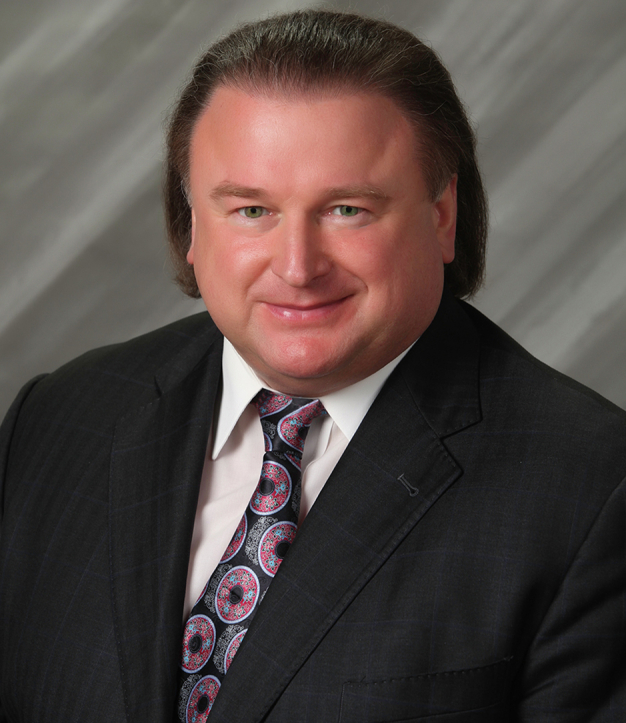

In this issue of *JACC: Advances*, Razavi et al^1^ make another significant contribution to our knowledge about the history of atherosclerosis. They demonstrate that almost half of 815 individuals enrolled in the MESA (Multi-Ethnic Study of Atherosclerosis) (average age 70.2 years initially) became positive for coronary artery calcification (CAC+) with a median time to conversion of 4.3 years. The main emphasis in their analysis was on which “nontraditional risk factors” were predictors of this conversion. Although albuminuria, carotid artery plaque, and thoracic aortic calcification did not improve discrimination of incident CAC when added to traditional risk factors, individually they were significant predictors. By contrast, apolipoprotein B (apoB), lipoprotein(a), high-sensitivity troponin T, and N-terminal probrain natriuretic peptide were not significant predictors, even individually. Thus, evidence of noncoronary atherosclerosis and nephropathy help to identify older asymptomatic patients who might benefit from CAC screening. The authors cite data demonstrating atherosclerotic cardiovascular disease (ASCVD) risk is higher in CAC+ vs CAC 0 individuals and suggest these positive markers are useful in identifying those at high risk for conversion and, therefore, constitute a subgroup who should receive intensive preventive therapy. We congratulate them on an analysis that has been carefully considered and meticulously done. Although we differ significantly in the conclusions we draw, we acknowledge that different interpretations of the same data are reasonable. Where you wind up often depends on where you are coming from.

We agree that, as a group, those with CAC+ are at higher risk of an ASCVD event than those with CAC 0. But CAC is a late manifestation of plaque evolution. Accordingly, the difference is between the risk of a group, all of whom have the disease and, therefore, all of whom may suffer the consequences of the disease and another group, only some of whom have the disease, and only these can suffer the consequences of the disease. We also agree that risk is an imperfect tool to identify those who could benefit from statin therapy to reduce ASCVD risk.^2^ However, when risk is sufficiently high as it was in this cohort—an average of 14.7%—statin therapy is undoubtedly cost-effective and indicated. It is clear from such clinical trials as the PROSPER (Prospective Study of Pravastatin in the Elderly at Risk) study^3^ and the HPS (Heart Protection Study)^4^ as well as meta-analyses^5^^,^^6^ that statin therapy reduces ASCVD events in patients 65 years of age and older in both the primary and secondary prevention settings. Given that age is the most significant driver of ASCVD risk, coronary imaging in most cases is not necessary to identify individuals likely to benefit from lipid-lowering therapy.

In our view, the most striking finding in this report was that just under half of all subjects who were CAC 0 at baseline became CAC+ after less than an average of 3.5 years of follow up. This is an extraordinary conversion rate in a short period of time. That the incidence of CAC+ rises sharply with age has been known for some time. However, these subjects were on average 70.3 years of age at baseline and, therefore, represent the group who had remained the exception to the rule, a group who might have been immune from atherosclerosis. They were not. In order for them to develop CAC during follow-up, they almost certainly had established coronary atherosclerosis at baseline. If half converted in such a short period of time, it seems certain that many more would convert with longer follow-up. Thus, atherosclerosis appears to be a relentless process that, given time, will appear and progress to an advanced form in the great majority of the population.

The primary objective of Razavi et al^1^ was to identify which nontraditional risk factors for ASCVD identified an increased risk of developing CAC in this older cohort. That thoracic artery calcification and carotid plaque were predictors of CAC development is not surprising given that they are forms of atherosclerotic disease. That microalbuminuria predicts CAC is not surprising given that microalbuminuria predicts ASCVD and increases risk for cardiovascular events. The pathophysiological relation of microalbuminuria to ASCVD is not obvious. Plasma apoB is not a significant predictor of microalbuminuria or impaired renal function. On the other hand, albuminuria is associated with increased hepatic very low-density lipoprotein production and secretion producing higher serum levels of apoB.^7^ Elucidating the pathophysiological basis for the relationship between microalbuminuria and increased ASCVD risk is a high priority.

A striking negative finding of this study was that apoB was only marginally higher in those who developed CAC compared to those who did not and that apoB did not significantly predict the likelihood of developing CAC. To be sure, a greater percentage of those who developed CAC were on lipid lowering therapy at baseline vs those who did not (21.7% vs 14.7%, *P* = 0.01) and there are no data on initiation of treatment during follow-up. Nevertheless, the finding is consistent with other studies demonstrating that the HRs of apoB as well as low-density lipoprotein-C and nonhigh-density lipoprotein-C decrease steadily with age.

Does this mean the apoB lipoproteins do not drive atherosclerotic disease in those who are older? Not at all. Trapping of apoB particles within the arterial wall is the root cause of atherosclerosis and the level of the apoB lipoproteins within plasma is the primary, but not exclusive, determinant of the number of apoB particles that will be trapped over time.^8^ Calcification is a histologic hallmark of advanced atherosclerotic disease. Thus, apoB causes atherosclerosis and CAC is a consequence of atherosclerosis. The declining HR of apoB with age, notwithstanding the ever increasing incidence of atherosclerotic disease with age, is due to the interplay of cause and consequence over time. Higher levels of apoB produce extensive advanced disease in shorter periods of time whereas lower levels of apoB produce extensive disease in longer periods of time. Atherosclerotic disease may appear and advance later in life with lower levels of apoB but, with time, appear and advance it will.

The total mass of atherosclerotic lesions is not unlimited because the area of the arterial wall is fixed and not all areas are equally susceptible to disease. Once extensive disease is present, it is the mass of disease that drives ASCVD risk, not the causes of more disease, such as apoB. Nevertheless, that the deposition of apoB particles continues to matter in those who are older has been established unequivocally by the positive impact of statin therapy on ASCVD risk in those who are older. Our hypothesis is that in those with established, extensive disease, the benefit of lowering apoB is not primarily due to a reduction in new lesion formation since so many lesions already exist but rather relates to promoting the stabilization or even healing/regression of established lesions that are not yet calcified and possible further stabilization of calcified plaques. Reducing deposition of cholesterol within the subendothelial space by lowering plasma apoB multiplies the efficiency of the physiological mechanisms which remove cholesterol from the arterial wall (eg, reverse cholesterol transport). Hence, although apoB is not predictive of atherosclerotic plaque calcification, reducing apoB with lipid-lowering therapies is beneficial.

Whatever the exact explanations, the fact that lowering apoB reduces risk even in those who are older with advanced disease establishes that apoB always matters. But apoB particles are not all that matters. Too little is known of the factors that govern the permeability of the endothelium to apoB particles and the factors that promote or reduce trapping of apoB particles that have entered the arterial wall.^9^ We note that although other studies have shown that increased serum levels of lipoprotein(a) correlate with increased risk for CAC and aortic valve calcification, this analysis from Multiethnic Study of Atherosclerosis did not.^10^ This will also require further study. As these authors have shown us, it is time to focus on the wall of arteries, not just on the concentration of atherogenic agents within the lumen of arteries. We have only begun to understand the complex relations between the causes of atherosclerosis and their complications within the arterial wall as affected by the passage of time.

## Funding support and author disclosures

Dr Toth has served on the Speakers Bureau for Amgen; and has been a consultant to Novartis. Dr Sniderman has reported that he has no relationships relevant to the contents of this paper to disclose.
